# Use of Magnetic Resonance Angiography in Diagnosis and Decision Making of Post-Traumatic, High-Flow Priapism

**DOI:** 10.1100/tsw.2008.35

**Published:** 2008-02-19

**Authors:** Ahmed El-Assmy, Ihab A. Hekal, Mohamed E. Abou-El-Ghar

**Affiliations:** ^1^ Urology Department, Urology and Nephrology Center, Mansoura University, Mansoura, Egypt; ^2^ Radiology Department, Urology and Nephrology Center, Mansoura University, Mansoura, Egypt

**Keywords:** MR angiography, high-flow priapism, perineal trauma

## Abstract

The ideal imaging modality should demonstrate the presence or absence of a clinically significant, causative vascular lesion that, in high-flow arterial priapism, may need intervention. We report a 22-year-old male with post-traumatic arterial priapism. Color Doppler ultrasound could not reliably identify a significant vascular lesion. Magnetic resonance angiography (MRA) demonstrated the presence of a cavernous artery pseudoaneurysm. Based on this finding, embolization was decided, with a successful outcome. Contrast-enhanced MRA appears to be a useful, noninvasive diagnostic tool for decision making in cases of high-flow priapism.

## INTRODUCTION

Priapism is a persistent penile erection that continues more than 4 h, unrelated to sexual stimulation. It is divided into ischemic (veno-occlusive, low flow) painful priapism and nonischemic (arterial, high flow) painless priapism[1]. Perineal or penile trauma is the most common cause of high-flow priapism (HFP), and this is usually due to an AV fistula or a pseudoaneurysm of the cavernosal vessels. Treatment for HFP varies from watchful waiting to mechanical compression with ice packs, embolization, or even open surgery with ligation of the artery concerned[2,3,4,5,6]. The choice of the optimal line of management depends on the presence or absence of a clinically significant, causative lesion that may need intervention. We describe the use of noninvasive imaging to facilitate the choice of an ideal approach for such a clinical dilemma.

## CASE REPORT

A 22-year-old male presented with painless priapism, 3 days after suffering a straddle injury. Physical examination revealed that the corporal bodies were turgid and not tender, while the glans and the corpus spongiosum were soft. Physical examination additionally revealed a scrotal hematoma. Diagnostic aspiration of the corpora cavernosa demonstrated bright red blood with high oxygen saturation. Penile color Doppler ultrasound (US) was carried out by an experienced radiologist with an Acuson-128 XP

(Acuson Corp., Mountain View, CA), with linear transducer 5–10 MHz. Doppler US showed increased peak systolic velocity (PSV) in the right cavernosal artery (43 cm/s) (Fig. 1). The PSV in the left cavernosal artery was 14 cm/s. The history, clinical examination, and Doppler sonography suggested arterial or high-flow priapism. However, the presence of a fistula or a pseudoaneurysm was not demonstrated by color Doppler US.

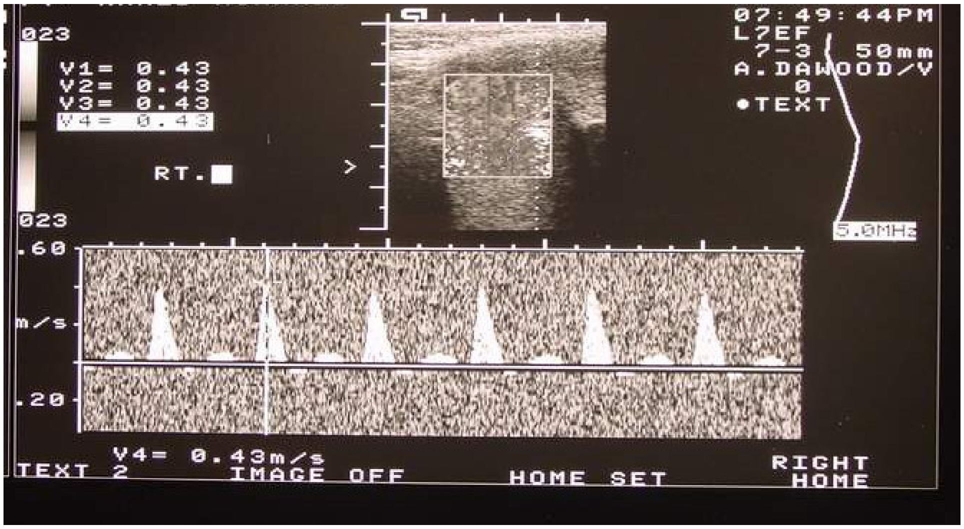

**FIGURE 1.** Color Doppler US of the proximal penis. PSV is high (43 cm/s), but a fistula or a pseudoaneurysm could neither be demonstrated nor excluded.

Contrast-enhanced magnetic resonance angiography (MRA) was performed and showed a right corpus cavernosum pseudoaneurysm (Fig. 2). The patient was scanned with a 1.5-T MRI scanner (SIGNA Horizon, General Electric Medical Systems, Milwaukee, WI) using body coil. Gadolinium-enhanced MRA was performed using intravenous injection of Gd-DTPA manually at a rate of 3–4/s with a dose of 0.3 mmol/kg body weight. Arterial and venous phases were scanned using automatic start scanning. Each phase lasted 12 s during breath hold and there was a 10-s interval between the two phases. The parameters of image scans were: TR = 3.8 ms, TE = minimum, flip angle= 20$^o$, band width = 62.5, slice thickness of 2.5 mm, and FOV = 36 cm.

Based on the MRA findings, the patient was considered for angiography and embolization**.** Angiography confirmed the presence of a pseudoaneurysm at the right corpus cavernosum (Fig. 3). The internal pudendal artery was embolized using 0.035-in. platinum microcoils (Boston Scientific, Boston, MA) and were delivered through the 6 French (F) angiographic catheter (Copra II, Terumo, Osaka, Japan). The postembolization picture showed no further filling of the pseudoaneurysm (Fig. 4). Complete detumescence of the penis occurred within minutes. Follow-up after 2.5 years revealed no abnormality, with good erectile function.

## DISCUSSION

Most cases (85%) of priapism appear to be of veno-occlusive origin, and this is termed low flow, ischemic, or painful priapism. In a minority of cases (15%), the cause is increased arterial inflow into the corpora cavernosa and is termed arterial or painless priapism[1]. Perineal or penile trauma is the most common cause of painless priapism, and this is usually due to an AV fistula or a pseudoaneurysm of the cavernosal vessels, circumventing the regulatory helicine arterioles. Venous outflow is not compromised,

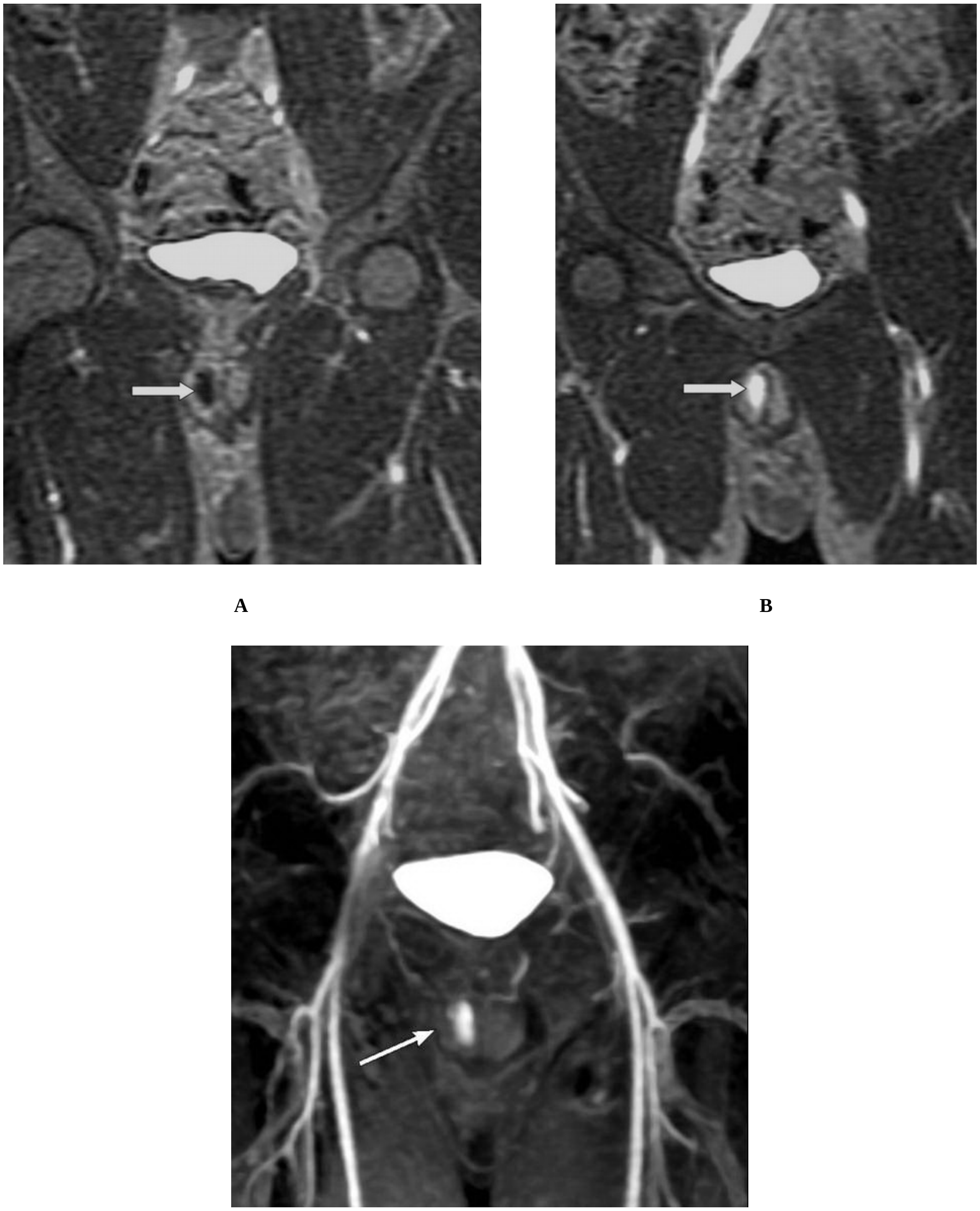

A B

C

**FIGURE 2.** Right cavernosal artery pseudoaneurysm. (A) Gd-MRA of the penis showing a hypointense saccular lesion at the right corpus cavernosum (arrow) at the early arterial phase; (B) at the venous phase, there is filling of the pseudoaneurysm (arrow); (C) at the reformatted MIP image, the relation of the pseudoaneurysm (arrow) with the cavernosal artery could be delineated.

tissue ischemia does not occur, and the condition remains painless[1]. Usually, there is a delay of a few days between the trauma and the onset of priapism, as in our patient, whose erection started 3 days following perineal trauma. This could be explained by formation of a clot that seals the injured artery early, which may be dislodged later during an episode of nocturnal tumescence.

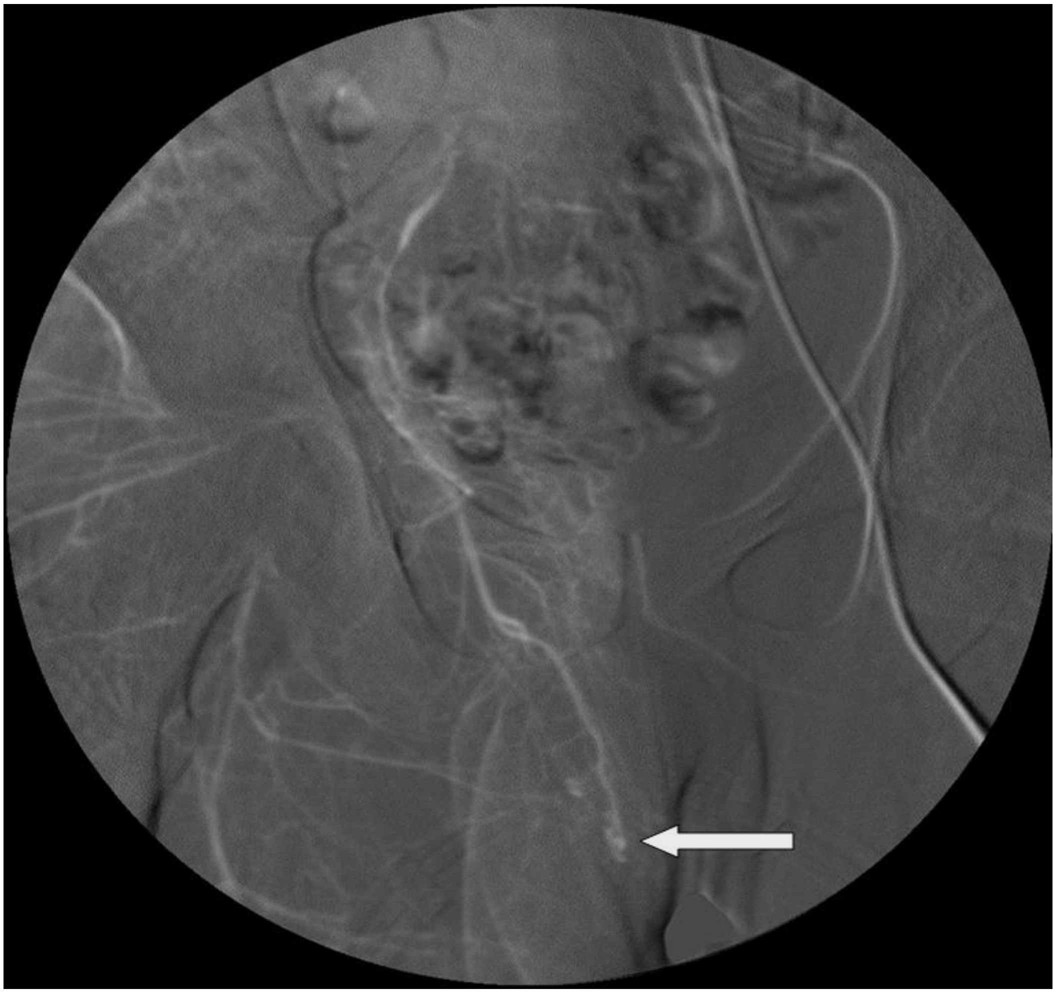

**FIGURE 3.** Pre-embolization subtraction arteriogram of the right iliac and pudendal arteries via left transfemoral approach showing an evidence of small-cavity-filled contrast (arrow) related to the right cavernosal artery.

Treatment for HFP varies from watchful waiting to arterial ligation or percutaneous embolization[2,3,4,5,6]. Some authors recommend watchful waiting as a first-line therapy in HFP as there is no tissue ischemia and it can resolve spontaneously without hazards. The disadvantages of watchful waiting are possible structural alterations resulting from excessive arterial inflow, which may lead to impotence as well as social and psychological difficulties related to the condition and should be discouraged[2].

The choice of an optimal therapeutic option for each patient may be confusing. The ideal imaging modality should demonstrate the presence or absence of a clinically significant, causative vascular lesion that may require intervention. Color Doppler US is currently considered the imaging modality of choice for diagnosis of HFP because it is sensitive, noninvasive, and widely available. The abnormal color Doppler patterns in high-flow arterial priapism are markedly increased flow within the corpus cavernosum, cavernosal artery, or a pseudoaneurysm[7,8]. Also color Doppler US can demonstrate extravasation of blood from the lacerated cavernosal artery as a characteristic color blush that extends into the erectile tissue. Besides the arterial-lacunar fistula, the penile vasculature can be evaluated to identify the feeding vessels[8].

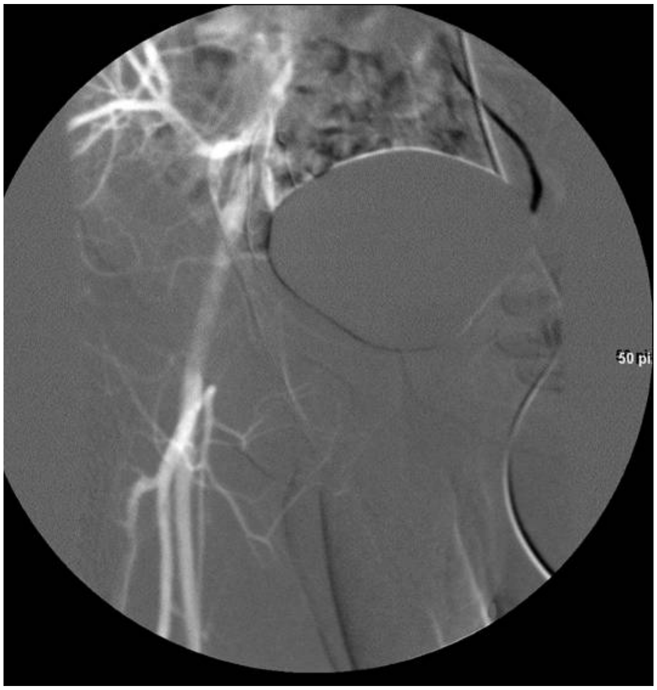

**A**

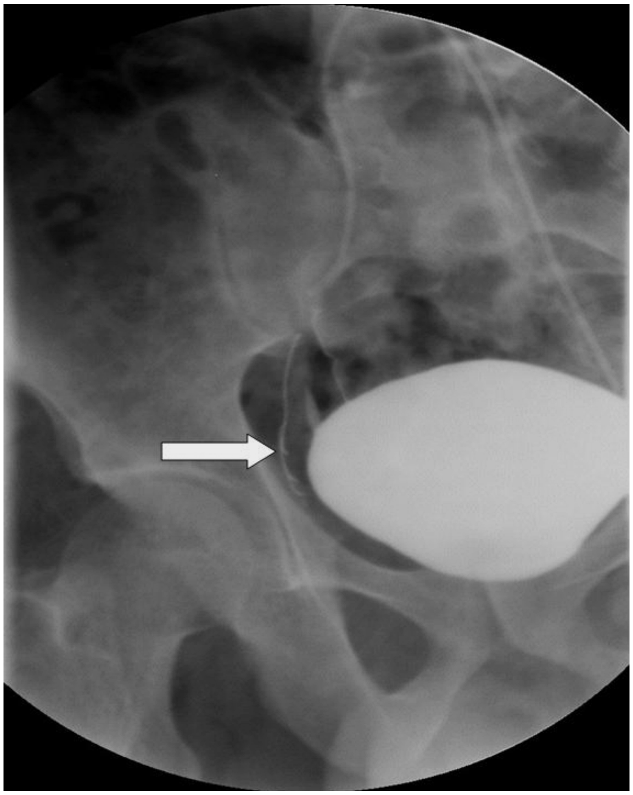

**B**

**FIGURE 4.** (A) Postembolization subtraction arteriogram showed complete closure of the lesion. (B) plain X-ray of the pelvis showing evidence of the platinum microcoils (arrow) in place.

However these findings were not reliably diagnosed or excluded in our patient. This is because color Doppler US has some limitations in diagnosing HFP; first, optimization of the color Doppler parameters to detect slow-velocity flow makes the color blush often display aliasing; in this situation, it can be difficult to identify the exact site of the cavernosal artery tear. In addition, owing to high intracavernosal pressure, extravasation of blood through the fistula can decrease, particularly during diastole, when the arterial pressure is lower[8].

In such a group of patients, with equivocal color Doppler findings, digital subtraction angiography remains the gold standard for diagnosis; however, it is an invasive procedure with possible complications.

MRI is useful for the investigation of penile disorders associated with veno-occlusive priapism[9]. To our knowledge, the MRI findings of cavernous artery pseudoaneurysm was reported previously once[9]. In the case described previously, the pseudoaneurysm was diagnosed by color Doppler US and the authors did not show any value for the use of MRA in their case[10]. Eracleous and associates described the use of MRA to diagnose the cause of post-traumatic HFP in a child in whom color Doppler US could not find a lesion; however, MRA also did not demonstrate any lesion[11].

In our patient, there was no identifiable lesion on color Doppler US. The presence of the pseudoaneurysm and its localization were detected with MRI. On the basis of the MRI findings, invasive intervention was recommended with a successful outcome.

An ideal imaging modality should demonstrate the presence or absence of a clinically significant causative lesion, which may need intervention in high-flow arterial priapism. Contrast-enhanced MRA appears to fulfill these requirements.
